# Whole organism and tissue-specific analysis of pexophagy in *Drosophila*

**DOI:** 10.1098/rsob.240291

**Published:** 2025-02-05

**Authors:** Francesco G. Barone, Marco Marcello, Sylvie Urbé, Natalia Sanchez-Soriano, Michael J. Clague

**Affiliations:** ^1^ Department of Biochemistry, Cell and Systems Biology, ISMIB, University of Liverpool, Liverpool L69 3BX, UK; ^2^ Centre for Cell Imaging, Institute of Systems, Molecular and Integrative Biology, University of Liverpool, Liverpool L69 7ZB, UK

**Keywords:** pexophagy, peroxisomes, mortalin, Hsc70-5, neurons, *Drosophila*

## Abstract

Peroxisomes are essential organelles involved in critical metabolic processes in animals such as fatty acid oxidation, ether phospholipid production and reactive oxygen species detoxification. We have generated transgenic *Drosophila melanogaster* models expressing fluorescent reporters for the selective autophagy of peroxisomes, a process known as pexophagy. We show that these reporters are colocalized with a peroxisomal marker and that they can reflect pexophagy induction by iron chelation and inhibition by depletion of the core autophagy protein Atg5. Using light sheet microscopy, we have been able to obtain a global overview of pexophagy levels across the entire organism at different stages of development. Tissue-specific control of pexophagy is exemplified by areas of peroxisome abundance but minimal pexophagy, observed in clusters of oenocytes surrounded by epithelial cells where pexophagy is much more evident. Enhancement of pexophagy was achieved by feeding flies with the iron chelator deferiprone, in line with past results using mammalian cells. Specific drivers were used to visualize pexophagy in neurons, and to demonstrate that specific depletion in the larval central nervous system of Hsc70-5, the *Drosophila* homologue of the chaperone HSPA9/mortalin, led to a substantial elevation in pexophagy.

**Figure:**
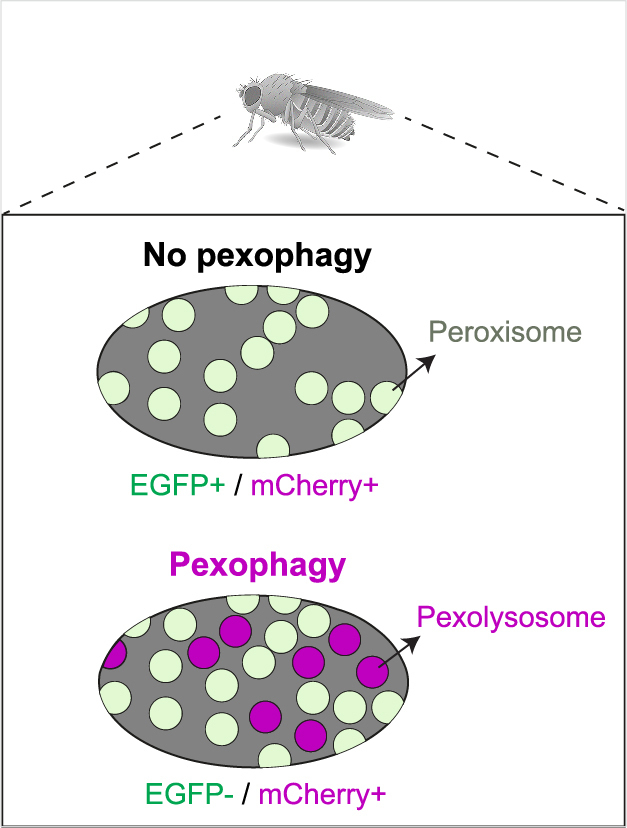

## 1. Introduction

Peroxisomes are discrete organelles that play key roles in lipid metabolism and reactive oxygen species (ROS) detoxification [1,2]. They are also essential for cell death by the ferroptosis pathway [3,4]. Their abundance is regulated via rates of biogenesis and division, on the one hand, and selective degradation by autophagy on the other [5,6]. This degradation process known as pexophagy can occur via different pathways, which involve either the acquisition of BNIP3L (hereafter referred to as NIX) or the binding of ubiquitin receptors such as NBR1, both of which bind to LC3 on autophagosomal membranes [7–9]. Diseases linked to peroxisome homeostasis and function, known as peroxisome biogenesis or Zellweger spectrum disorders are profound multisystem diseases with severe effects on the central nervous system, for which it has been suggested that defective pexophagy is a fundamental feature [10,11].

The distribution and size of peroxisomes within both cells and organisms have been studied by electron microscopy and more recently by the expression of green fluorescent protein (GFP)-tagged peroxisomal markers [12–14]. This has allowed depletion of peroxisomes to be linked to disease-associated mutations but does not formally distinguish between reduced biogenesis versus enhanced pexophagy. A fluorescence approach to measuring selective autophagy entails expressing a pH-sensitive ratiometric reporter protein that localizes to the compartment in question. When these probes are delivered to acidic endolysosomal compartments, changes in their fluorescent properties can be visualized. For example, two probes, mito-QC and mito-mKeima have been developed to measure the selective autophagy of mitochondria, known as mitophagy [15,16]. We and others have substituted an SKL import signal, which confers irreversible translocation into the peroxisomal matrix, to generate reporters for measuring pexophagy in mammalian cells [7,8,17,18]. The ability to visualize mitophagy in intact organisms, such as mice or flies, has provided insight into the tissue variation and changes during development. Analysis of mitophagy in PINK1 or Parkin mutant animals proved critical to the realization that the majority of basal mitophagy is independent of these linked genes [19,20].

Peroxisomes in *Drosophila* have been analysed using the expression of GFP-reporters in genetic screens of peroxisomal size or number [13,21,22]. Here we have extended this approach to directly visualize the magnitude of pexophagy alongside these parameters, by adopting both pexo-QC and pexo-Keima reporters. Furthermore, we take advantage of light sheet microscopy to capture the distribution of pexophagy across developmental stages of the whole organism in a single image. As a first application of these models, we have performed imaging experiments on whole larvae to examine the impact of Hsc70-5 (mortalin) depletion upon pexophagy [22].

## 2. Results

### 2.1. Characterization of pexophagy reporter flies

**Figure 1.**
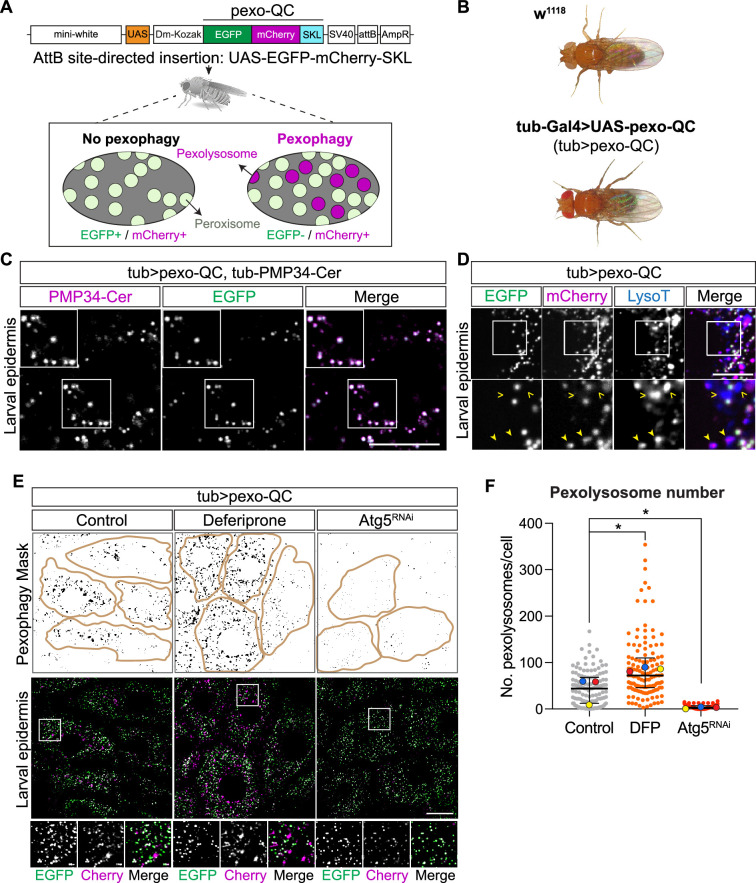
Generation of the pexo-QC *Drosophila melanogaster* for *in vivo* detection of pexophagy and peroxisomal abundance. (A) Diagram of the cassette used for expression of an EGFP-mCherry-SKL fusion protein (pexo-QC) to create the pexo-QC *Drosophila* (elements not to scale). This includes a mini-white gene, upstream activating sequence (UAS), a *D*. melanogaster Kozak sequence (Dm-Kozak), the EGFP-mCherry-SKL coding sequence, an SV40 polyadenylation sequence, an attB site for site-directed insertion and an ampicillin resistance gene (AmpR). At neutral pH, peroxisomes display both GFP and Cherry fluorescence, whereas only Cherry fluorescence is retained at the acidic pH of the lysosome (pexolysosomes, magenta). (B) Bright field images of *w^1118^* flies and *tub-Gal4>UAS-pexo-QC* flies (9–12 days old), indicating no observable phenotypical changes or gross morphological abnormalities in the pexo-QC *Drosophila* model. (C) High-resolution Airyscan live images of L3 larval epidermis comparing EGFP fluorescence signal (green) of pexo-QC with exogenous expression of peroxisomal membrane protein PMP34-Cer (*Tub-PMP34-Cerulean*, shown in magenta) representative of three independent experiments, with at least three animals per condition in each experiment. Scale bar: 20 μm. (D) Confocal live imaging analysis of pexo-QC larval epidermal cells costained with LysoTracker to identify lysosomes (blue). Representative of three independent experiments, with at least three animals per condition in each experiment: >LysoTracker^(+)^, mCherry^(−)^ puncta; filled arrowhead, LysoTracker^(+)^/mCherry^(+)^ puncta. Scale bar: 10 μm. (E) Representative confocal images of pexo-QC larval epidermal cells upon expression of an *Atg5*-RNAi transgene (*Atg5^RNAi^*) to inhibit autophagy or exposure to the iron chelator deferiprone (DFP, 65 µM) to induce pexophagy. Scale bar: 20 μm. Also shown is a pexophagy mask, automatically generated from the mCherry/GFP ratio using the Fiji macro previously reported [23]. (F) The graph shows the number of pexolysosomes per cell for each condition. The mean and s.d. of three colour-coded independent experiments are shown; one-way ANOVA with Dunnett’s multiple comparison test for three colour-coded independent experiments with a minimum of three animals per condition in each experiment. *p < 0.05. Genotypes analysed were *w^1118^; UAS-pexo-QC/+; tub-Gal4/tub-PMP34-Cer* (C), *w^1118^; UAS-pexo-QC/+; tub-Gal4/+* (D) and *w^1118^; UAS-pexo-QC/+; tub-Gal4/+, w^1118^; UAS-pexo-QC/UAS-Atg5^RNAi^; tub-Gal4/+ *(E).

We started by making Gal4/UAS-inducible transgenic *Drosophila* strains that express either EGFP-mCherry-SKL (hereafter pexo-QC) or mKeima-SKL (hereafter pexo-Keima) pexophagy reporters, which were chosen for their ability to detect pH changes within autophagic structures (figure 1A,B; electronic supplementary material, figure S1A). Upon fusion between the autophagosome and an acidic lysosome, the EGFP fluorescence from the pexo-QC is lost, owing to the destabilization of the protein. The more stable mCherry fluorescence remains, leaving red puncta characteristic of pexolysosomes (here pseudocoloured as magenta). In the case of pexo-Keima, the acidic conditions result in a shift in the excitation spectrum of the fluorophore. For each reporter, multiple lines with equivalent expressions were developed, and subsequently a single line was selected for further analysis. We observed that the ubiquitous expression of these reporters, using a Tubulin Gal4 driver (*tub-Gal4*), had no apparent impact on development or viability, suggesting that they were well tolerated by the organism. To verify the expected targeting to peroxisomes, we examined colocalization with the peroxisomal membrane protein PMP34 in larval epidermis. We found that both pexo-QC and pexo-Keima reporter colocalize well with peroxisomes (PMP34-Cer positive organelles) in the green pH-neutral channel as expected (figure 1C; electronic supplementary material, figure S1B). The first indications of pexophagy are evidenced by magenta puncta (derived from pexo-QC) that colocalize with the marker of acidic endolysosomal compartments, LysoTracker (figure 1D). Similarly, pexo-Keima’s fluorescence excitation spectrum shifts at low pH, with an enhanced signal from excitation at 561 nm (electronic supplementary material, figure S1A). We next quantitated the number of pexolysomes per cell following two experimental interventions. The iron chelator deferiprone (DFP) is known to induce pexophagy in mammalian cells by inducing expression of the pexophagy adaptor molecule BNIP3L/NIX [8,9]. Here we show that its administration to flies, via a feeding regimen, produces a similar effect (figure 1E,F). We also expressed an RNAi transgene suppressing the core component of the autophagy machinery, Atg5, which strongly reduced the pexophagy levels (figure 1E,F). Qualitatively comparable results were found with pexo-Keima flies (electronic supplementary material, figure S1C). In all subsequent experiments, we have used pexo-QC expression as it showed superior brightness and has the advantage of being fixable.

### 2.2. A whole organism view of pexophagy gained via light sheet microscopy

**Figure 2.**
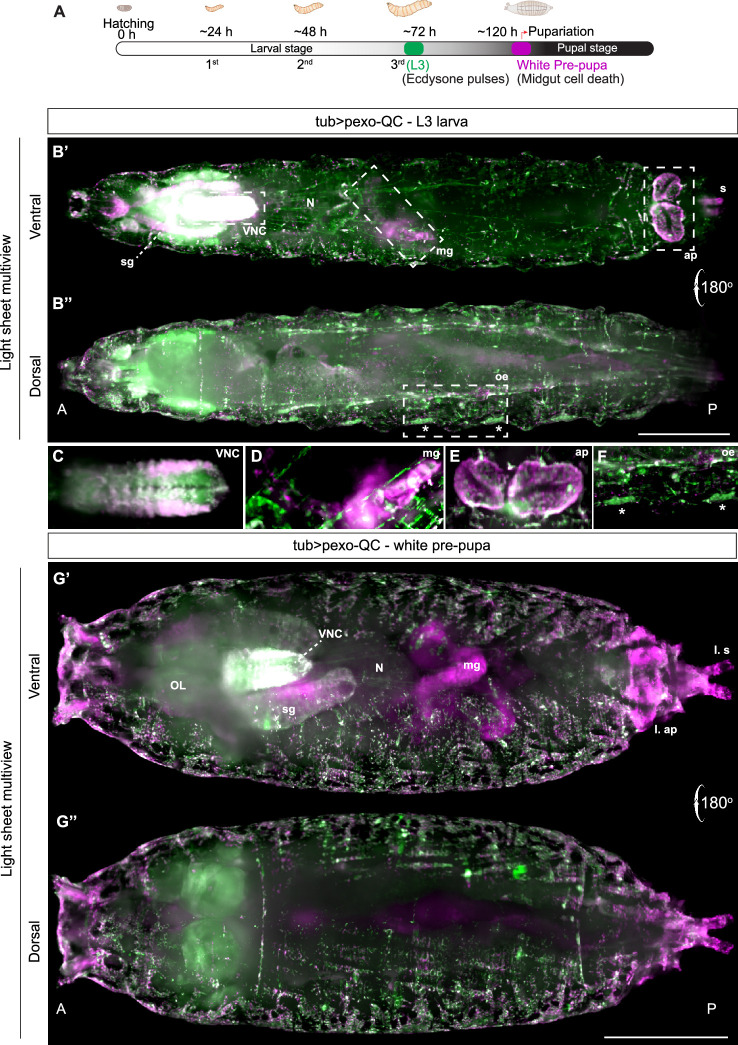
Light sheet microscopy reveals tissue-specific distribution of pexophagy in intact *Drosophila* larvae and pre-pupae. (A) Schematic representation of *Drosophila* embryo, larval development and early pupariation stage (white pre-pupa). (B',B'') Light sheet fluorescence microscopy (LSFM) multiview images of fixed pexo-QC L3 larva (*tub>pexo* QC), ventral view (B') and dorsal view (B''). (C–F) Enlarged insets of the specimen shown in (B') and (B''). Z-thickness and image contrast settings were adjusted to highlight the differential distribution of peroxisomes and pexolysosomes in the respective tissues. (C) Pexophagy in the ventral nerve cord (VNC) with heterogeneous pexophagy levels. (D) Enrichment of pexolysosomes in a portion of the larval midgut (mg). (E) Enrichment of pexolysosomes in the larval anal plates (ap). Insets shown in (D) and (E) were rotated by −45° and −90°, respectively, for improved visualization and clarity. (F) Peroxisomal enrichment in larval oenocyte (oe) clusters. Asterisks indicate two individual oenocyte clusters. Salivary glands (sg); nerves (n). (G',G'') LSFM multiview images of white pexo-QC pre-pupae (tub>pexo QC), ventral view (G') and dorsal view (G''). Optical lobes (OL); salivary glands (sg); larval anal plates (L. ap); larval spiracles (L. s). The genotype analysed was *w^1118^; UAS-pexo-QC/+; tub-Gal4/+*. Scale Bar 500 μm. Single channel images of (B',B'') and (G',G'') are shown in electronic supplementary material, figures S3 and S4, respectively.

**Figure 3.**
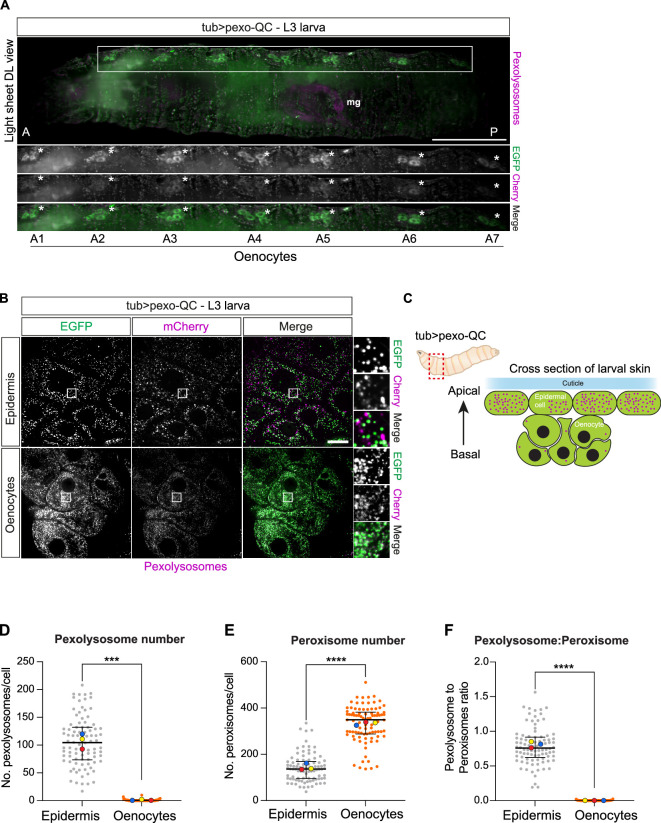
*Drosophila* oenocytes are enriched with peroxisomes but show low levels of pexophagy. (A) A representative Z-stack maximum intensity projection image acquired from a dorsolateral (DL) view of pexo-QC L3 larvae (*tub > pexo* QC) using a light sheet microscope reveals seven identifiable peroxisome-rich clusters (white asterisks, *A1–A7*) that based on their localization and morphology, can be classified as oenocytes. Scale bar: 500 μm. (B) Representative confocal images comparing pexophagy levels between oenocytes and the surrounding epidermis in living pexo-QC larvae using the same driver (tub-Gal4). Images were acquired using a 3i confocal microscope with a 63× NA 1.4 objective lens. Scale bar: 20 μm. (C) Schematic representation of a cross section of the larval skin in *Drosophila melanogaster*, showing the relative positions of epidermal cells, rich in pexolysosomes (shown in magenta) and oenocytes that have few pexolysosomes. Genotype analysed was *w^1118^; UAS-pexo-QC /+; tub-Gal4/+.* (D–F) Graphs show the number of pexolysosomes and peroxisomes per cell as well as their respective ratios. At least 30 cells from a minimum of three animals were analysed per condition in each of the three independent experiments. The mean and s.d. of three colour-coded independent experiments are indicated. Statistical significance was determined by unpaired *t*‐test; ***p < 0.001, ****p < 0.0001.

We employed light sheet microscopy, alongside spinning disc confocal microscopy, to visualize the whole organism at different stages of development, using the tubulin Gal4 driver for ubiquitous pexo-QC expression. Pexophagy was evident across the organism, but unevenly distributed, with marked hot spots discernible by confocal microscopy in the living late-stage embryo (electronic supplementary material, figure S2). We also employed fixation prior to visualization of third instar larvae (hereafter L3) and white pre-pupae by light sheet microscopy (figure 2; electronic supplementary material, figures S3, S4). Highly elevated areas of pexophagy are evident within the central nervous system, anal plate and mid-gut (where autophagy plays a role in cell death associated with developmental remodelling; figure 2B–F) [24]. Overall, pexophagy is most prominent in the pre-pupae (figure 2G). There are also prominent areas rich in peroxisomes but low in pexophagy including seven peroxisome-rich, pexophagy poor clusters comprising 4−6 cells located in a basal position below the epidermis (figure 3). On the basis of this signature distribution and cell size, these are highly likely to represent oenocytes, which are large secretory, hepatocyte-like cells in *Drosophila* and the major sites for very long chain fatty acid synthesis [25,26]. In line with this function, they are known to be sites of peroxisome enrichment [27,28]. Our data clearly indicate that peroxisomes exhibit a very low turnover rate in this organ, which we propose underlies their enrichment. In contrast, the prospective oenocytes are surrounded by epidermal cells, which show significantly higher levels of pexophagy (figure 3B–F).

### 2.3. Visualization of pexophagy within the central and peripheral nervous systems

**Figure 4.**
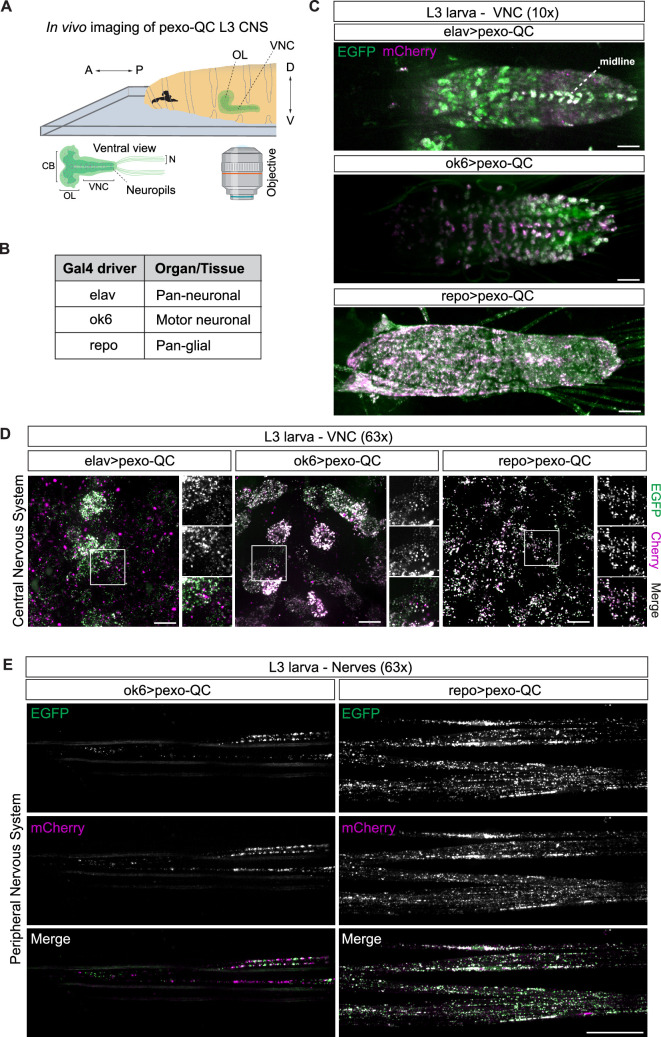
Visualization of neuronal and glial pexophagy (*in vivo*) in the nervous system of *Drosophila* larvae. (A) The imaging set-up utilized to explore *in vivo* pexophagy in the nervous system of pexo-QC L3 larvae using a 3i spinning disc confocal microscope is depicted schematically. Top: position of larvae immobilized in 10% chloroform halocarbon oil. Bottom: ventral view of the sample’s central and peripheral neural systems, ventral nerve cord (VNC) and nerves (N), respectively. A: anterior; P: posterior; D: dorsal; V: ventral; CB: central brain; OL: optical lobes. (B) Table displaying the different neural drivers employed in the study to analyse pexophagy in the pexo-QC larvae’s nervous system: elav-Gal4, a pan-neuronal, ok6-Gal4, a motor neuronal and repo-Gal4, a pan-glial driver. (C) Representative maximum intensity projection images of confocal Z-stacks of the VNC of living pexo-QC larvae acquired using the imaging set-up described in (A). Images were acquired using a 10× NA 0.3 objective. Scale bar: 50 μm. (D) Higher magnification images of maximum projection images of confocal Z-stacks of the VNC of live pexo-QC larvae using a 63× NA 1.4 oil immersion objective. Scale bar: 10 μm. (E) Representative confocal maximum intensity projection images of confocal Z-stacks of nerves in live pexo-QC larvae using distinct neural drivers. Left. *in vivo* pexophagy in motor neurons (*ok6-Gal4*). Right. *in vivo* pexophagy in wrapping glial cells (repo-Gal4). Scale bar: 20 μm. Genotypes analysed were *w^1118^; UAS-pexo-QC/+; elav-Gal4/+* (neurons), *w^1118^; UAS-pexo-QC/ok6-Gal4; +/+* (motor neurons), *w^1118^; UAS-pexo-QC/+; repo-Gal4/+* (glial cells).

**Figure 5.**
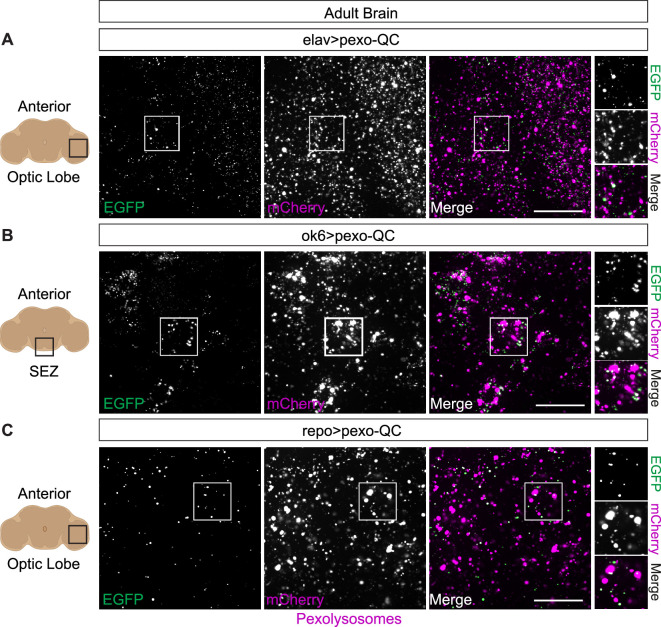
Pexophagy (*ex vivo) in* neuronal and glial cells in the adult central nervous system of pexo-QC flies. Representative confocal images of live pexo-QC adult brains from 9−12 day old flies, using distinct neural drivers (3i spinning disc confocal microscope, 63× NA 1.4 objective). (A) Neuronal pexophagy in the optical lobe of *ex vivo Drosophila* pexo-QC adult brain (*elav > pexo* QC). (B) *Ex vivo* pexophagy in the motor neurons of the suboesophageal zone of *Drosophila* pexo-QC adult brain (*ok6 > pexo* QC). (C) Glial pexophagy in the optical lobe of *ex vivo Drosophila* pexo-QC adult brain (*repo > pexo* QC). Genotypes analysed were *w^1118^; UAS-pexo-QC/+; elav-Gal4/+* (neurons) (A), *w^1118^; UAS-pexo-QC/ok6-Gal4; +/+* (motor neurons) (B), *w^1118^; UAS-pexo-QC/+; repo-Gal4/+* (glial cells) (C). Scale bar: 20 μm.

The sheer density and complexity of the central nervous system require specific drivers to visualize different cell populations *in vivo*. To provide a first qualitative glimpse of pexophagy distribution within the central nervous system, we have separately expressed pexo-QC under the control of pan-neuronal (*elav-Gal4*), motor neuronal (*ok6-Gal4*) and pan-glial (*repo-Gal4*) drivers (figure 4A,B). It is also clear that at the third instar larval stage, the cell bodies of motor neurons show much higher levels of pexophagy than the total neuronal population, with high pexophagy apparent along either side of the midline (figure 4C,D). In addition, in the peripheral nervous system under high magnification, individual pexo-QC puncta can be visualized in the axonal projections of motor neurons, which exhibit a high degree of pexophagy (figure 4D). In the glia, surrounding the nerves, the density of peroxisomes is higher than that of motor neurons, and the evidence for pexophagy is correspondingly lower (figure 4E). Visualization of the adult *Drosophila* brain is more challenging and requires dissection prior to ‘live-cell’ imaging experiments. We found pexophagy to be prominent in the optic lobe (neurons and glia) and for motor neurons in the suboesophageal zone (figure 5).

### 2.4. Direct testing of Hsc70-5 gene function in the pexophagy pathway

**Figure 6.**
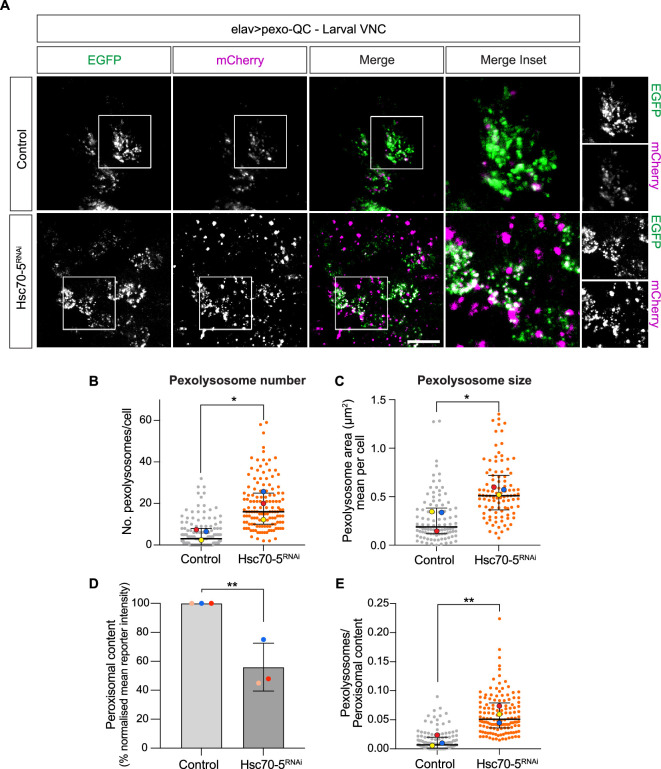
Hcs70-5 depletion enhances *in vivo* basal pexophagy in the ventral nerve chord (VNC) of L3 larvae. (A) Representative Z-stack maximum intensity projection (live) images of *in vivo* larval VNC neurons (elav > pexo QC) ± Hcs70-5 depletion (*Hcs70−5^RNAi^*). (B–E) Graphs show the number and mean area (size) of pexolysosomes per cell as well as peroxisomal content (percentage of normalized mean reporter intensity, EGFP) and the pexolysosome to peroxisomal content ratio. At least 30 cells from a minimum of three animals were analysed per condition in each independent experiment. The mean and s.d. of three colour-coded independent experiments are indicated. Statistical significance was determined by unpaired *t*‐test; *p < 0.05, **p < 0.01. Scale bars 10 μm. Genotypes analysed were *w^1118^; UAS-pexo-QC/+; elav-Gal4/+, w^1118^; UAS-pexo-QC/UAS-Hcs70−5^RNAi^; elav-Gal4/+.*

Mortalin/HSPA9 was identified as a pexophagy regulator and shown to partially localize to peroxisomes, where it has been proposed to suppress the generation of pexophagy-promoting ROS [22]. Reduction of this protein has been linked to neurodegenerative diseases such as Parkinson’s and Alzheimer’s [29–31]. Jo *et al.* have shown a loss of GFP-tagged peroxisomes in flies following depletion of the mortalin *Drosophila* homologue Hsc70-5, but did not directly visualize pexophagy, nor investigate its role in the central nervous system [22]. Here we use *in vivo* imaging of pexo-QC to confirm a strong increase in pexophagy (> fivefold) in the larval ventral nerve cord following Hsc70-5 depletion with a previously validated RNAi line against *Hsc70-5 (UAS-Hsc70-5^RNAi^* [22]; figure 6). These data confirm that *Drosophila* Hsc70-5 is a suppressor of pexophagy and further validate our pexo-QC line as an efficient tool to measure pexophagy.

## 3. Discussion

The introduction of selective autophagy reporters into whole organismal models has been applied to nematodes, flies, zebrafish and mice [16,19,32–34]. Each organism offers different pros and cons. For flies, the advantages include a relatively simple anatomy and low husbandry costs. Here, we also highlight their suitability for light sheet imaging of the entire fly larvae and pre-pupae, providing the first whole organism overview of any form of selective autophagy. The pexo-QC and pexo-Keima constructs are unlikely to perturb peroxisome dynamics as they simply couple a three amino acid import sequence to metabolically inert fluorophore proteins that are sequestered within the peroxisomal matrix. They provide tools for visualizing the terminal stages of pexophagy, wherein the autophagosome has fused with the lysosomal membrane to generate a so-called pexolysosome. For pexo-QC, these structures are characterized by their red (here pseudocoloured magenta) fluorescent signal alongside the absence of green fluorescence, owing to the instability of GFP in the prevailing acidic environment. In some of the neuronal images presented, there are certain puncta that appear to be green only. This is consistent with images of *Drosophila* brain using a corresponding non-selective autophagy reporter [35] and is a reflection of levelling procedures to accommodate high-intensity red puncta without image saturation.

Mitophagy has received particular attention and its visualization combined with genetic manipulation has allowed assessment of the contribution of different molecular mechanisms in the context of different organisms [19,20,32]. For example, the PINK1/Parkin pathway has been definitively shown to contribute little to the bulk turnover of mitochondria in flies and mice [19,20]. Peroxisomes and mitochondria cooperate in the metabolism of cellular lipids and ROS, in fact sharing many proteins [36]. In mammalian cells, pexophagy and mitophagy may proceed through a NIX/BNIP3-dependent pathway that links the respective membranes to autophagosomes [8,9,37–39]. It has been proposed that their turnover may be coupled via the regulation of NIX, but different modes of NIX regulation at each organelle can exist. We envision a future study wherein our pexo-QC fly can be directly compared with the mito-QC model to investigate this issue, assessing the role of the fly homologue of NIX/BNIP3.

The introduction of any new fly reporter model enables a genetic strategy to test literature claims of gene function. Our finding that reduction in Hsc70-5 enhances pexophagy confirms the model of Jo *et al.* [22], who used indirect assays to infer this effect. Thus, in the present study, we have demonstrated both chemical (DFP) and genetic enhancement of pexophagy. The ability to identify tissues showing distinct pexophagy profiles within an organism is exemplified by our finding that prospective oenocytes show extremely low levels of pexophagy while being replete with peroxisomes. As peroxisomes play especially important roles in this tissue, to effect very long chain fatty acid synthesis, it is interesting to note their correspondingly privileged protection from turnover [25]. One possible mechanism would be suppression of the fly homologue of NIX/BNIP3, CG5059, but this is not imposed at the transcriptional level, according to the relative mRNA levels reported in Fly Cell Atlas project data [8,9,40].

In summary, we introduce a tool for pexophagy research that will provide the field with new opportunities to assess gene function and metabolic control mechanisms. Our flies add to the suite of organismal models for selective autophagy and open up opportunities for direct comparison with those that use the same reporter type (organelle-QC).

## 4. Material and methods

### 4.1. *Drosophila* stocks, husbandry and reagents

Flies were maintained on standard fly food at 25°C in the common cornmeal-agar medium (390 g glucose, 360 g maize, 250 g yeast, 40 g agar, 135 ml 10% Nipagen, 15 ml propionic acid in 5 l of water). Fly stocks were obtained from BDSC (indicated with BL no.), VDRC, FlyORF (indicated with F no.), NIG or as otherwise indicated. For full listings of genotypes, see electronic supplementary material, table S1. Stocks used in this study are *w^1118^* [41] and *tub-Gal4 III* [42], *ok6-Gal4 II* (a gift from C. O’Kane [43]), *Repo-Gal4 III* [44], *elav-Gal4 III* [45], *UAS-atg5 RNAi II* (VDRC, 104461), *UAS-pexo-QC II* (this study), *UAS-Keima-SKL II* (this study), *UAS-Hsc70-5 RNAi KK* (VDRC, KK106236), *tub-PMP34-Cerulean III* (BL64246). All larvae shown in this study were actively feeding at the time of collection unless otherwise indicated. To administer DFP, pexo-QC and pexo-Keima (mKeima-SKL) L2 larvae were starved for 4 h and then transferred to a tube containing a mixture of water, 5% sucrose and 65 µM DFP. After a 20 h incubation period, L3 were selected and imaged to measure pexophagy levels. Drug administration in *Drosophila* larvae and DFP concentration are adapted from Kim *et al*. [46] and Lee *et al.* [19]*,* respectively. All reagents were purchased from Sigma Aldrich unless otherwise stated.

### 4.2. Transgenic line construction

A pexo-QC construct (pcDNA3.1-EGFP-mCherry-SKL) was generated by PCR amplifying pEGFP from pEGFP-C1 and inserting this into pcDNA3.1-mCherry-SKL (addgene, no. 54520). Also, pcDNA3.1-EGFP-mCherry-SKL was inserted in a pUAST.attB (DGRC, no. 1419) destination vector for specific phiC31-based transgenesis. For the pexo-Keima line, Keima-SKL was PCR-amplified from pcDNA3.1-Keima-SKL-Neo and cloned into the *Drosophila* expression vector pUAST.attB to generate the pUAST.attb-Keima-SKL construct [17]. Both pUAST.attB-EGFP-mCherry-SKL (pexo-QC) and pUAST.attb-Keima-SKL (pexo-Keima) constructs were verified by sequencing before sending for phiC31-mediated transgenesis at the *Drosophila* Research Facility, University of Manchester. Plasmids were integrated into attP40 sites. Several transformant lines were examined for consistency before settling on a single line for future investigation.

### 4.3. Lysotracker assay

*Drosophila* larval epidermis was dissected in phosphate-buffered saline (PBS) and incubated for 10 min with 0.7 mM LysoTracker Deep Red (Invitrogen). Dissected tissues were immersed in PBS on glass slides, covered and imaged immediately with a 3i spinning disc confocal fluorescence microscope (Intelligent Imaging Innovations, Germany, SlideBook 3i v. 3.0) equipped with a 63× /NA 1.4 oil objective and a scientific complementary metal-oxidase-semiconductor (sCMOS) camera, Hamamatsu. The protocol was adapted from Lee *et al.* [19].

### 4.4. *Drosophila* pexo-QC embryo preparation and imaging

The protocol was adapted from previous publications [47,48]. Late-stage 16 embryos of the desired genotypes were collected at 22°C and dechorionated by incubating in a solution of 50% bleach and 50% water for 1.5 min, followed by extensive rinsing in water to stop the reaction. The dechorionated embryos were then washed in ddH_2_O and spread on a fresh 2% agar/apple juice plate. Late-stage pexo-QC-positive (green+/red+) embryos were harvested using a Leica MZ10F-FLUO fluorescent microscope. Only embryos lacking the chorion (no dorsal appendices) and with an intact vitellin outer layer were selected. After collection, the pexo-QC embryos were immediately transferred onto an embryo chamber, consisting of a coverslip attached to double-sided sticky tape forming a channel filled with halocarbon oil 700 (Sigma; H8898−50ML [47]). The living embryos were spaced appropriately within the channel and covered by another coverslip before being imaged using a spinning disc confocal microscope (3i, Intelligent Imaging Innovations, Germany, SlideBook 3i v. 3.0) equipped with a 10× NA 0.3 objective lens and a sCMOS camera, Hamamatsu.

### 4.5. Light sheet imaging and sample preparation of whole-body *Drosophila* larvae and white prepupae

Digestion and fixation steps of both L3 and white pre-pupae were adapted from Pende *et al*. [49]. Briefly, samples were treated at 37°C for 45 min with 0.03% proteinase K (Sigma, no. P2308-10MG) in pre-warmed PBS to promote digestion of the superficial layer of larvae and pre-pupae. After 3× washes in PBS at room temperature for 10 min, samples were fixed in 4% paraformaldehyde at 4°C overnight followed by 3× washing with PBS at room temperature for 30 min. After the washing steps, animals were immediately mounted on a glass capillary tube (Brand GmbH, Wertheim, Germany) for light sheet imaging. To do that, 1% agarose (w/v; Agarose low melt, Roth, Germany) was melted in a heat block at 82°C. The melted agarose was allowed to cool to approximately 50°C. A drop was poured on the *Drosophila* samples and the mixture was sucked into a glass capillary tube, allowed to solidify and visualized in a Z.1 Light-Sheet microscope (Zeiss, Germany) with a 5×/0.1 illumination objective and an EC Plan Neofluar 5×/0.16 detection objective, using Zen software (Zeiss, Germany) for image acquisition and processing. Images were acquired in multiview with 0° and 180° angles (ventral and dorsal sample sides) using a pco.edge sCMOS camera (PCO, Germany). Images acquired using two-side illumination were automatically fused. For each multiview, a Z-stack maximum intensity projection was generated using Fiji v. 2.9.0 (ImageJ [50]). The light sheet analysis was performed for three independent experiments.

### 4.6. Colocalization of peroxisomal markers

For pexo-QC (EGFP channel) and PMP34-Cer co-localization in the larval epidermis, the tissues were imaged immediately after dissection, and samples were imaged using a Zeiss LSM900 with Airyscan (63×/NA 1.4 oil; Zen Blue software). For pexo-Keima (mKeima-SKL) samples, colocalization was performed with dissected unfixed larval epidermis using a 3i Marianas spinning disc confocal microscope (63×/ NA 1.4, Hamamatsu sCMOS camera; Slide Book 3i v.3.0 software).

### 4.7. Pexophagy analysis

For *in vivo* nervous system pexophagy studies, living L3 larvae were immobilized in halocarbon oil containing 10% chloroform and mounted on a glass slide with a top coverslip (figure 4A). Adult *Drosophila* were immobilized on ice. Thereafter, brains were carefully dissected in Dulbecco’s PBS (Sigma, RNBF2227) utilizing Dumont no. 5 Forceps (Dumont no. 5, no. 11295-10) and delicately placed in MatTek 35 mm dishes (MatTek, no. P35G-1.5-14-C) filled with PBS. A coverslip was firmly attached to the dish using double-sided tape. *In/ex vivo Drosophila* sample images were acquired using a 3i Marianas spinning disc confocal microscope (10× air NA 0.3, 40× oil NA 1.3 and 63× oil NA 1.4 objectives, Hamamatsu sCMOS camera; Slide Book 3i v. 3.0 software, 25°C incubator). Images were acquired sequentially using the following settings: pexo-QC samples: 488 nm laser, 525/30; 561 nm laser, 617/73 nm emission, Keima-SKL samples 445 nm laser, 617/73 nm emission; 561 nm laser, 617/73 nm emission. Pexophagy levels in pexo-QC samples were determined using the semi-automated ‘mito-QC Counter’ plugin implemented in Fiji v. 2.9.0 software as previously described [23,51]. For peroxisomal count, images were thresholded and the number of ‘green puncta’ was determined using the ‘analyse particle’ function implemented in Fiji [50]. The analysis of pexophagy involved three independent experiments, with at least three animals per condition acquired in each experiment.

### 4.8. Statistical analysis

Values of *p* are calculated using the unpaired *t*‐test, one-way ANOVA and Dunnett’s multiple comparisons post hoc test, and are denoted as *p < 0.05, **p < 0.01, ***p < 0.001 and ****p < 0.0001. GraphPad Prism 9 was used for all statistical analyses.

## Data Availability

Raw data for experiments presented can be furnished on request. Files will be deposited in a fly repository. Supplementary material is available online [52].
